# Research on the mechanism of Duhuo in treating intervertebral disc degeneration based on bioinformatics and network pharmacology

**DOI:** 10.1097/MD.0000000000048991

**Published:** 2026-06-05

**Authors:** Qian Yan, Fei Liu, Zhiwei Xu, Chi Zhang, Lei Yang, Xiaofei Wu, Chen Jiang, Feng Chen, Zhifa Li

**Affiliations:** a Department of Orthopedics, The Second Affiliated Hospital of Guangxi University of Chinese Medicine, Nanning, China; b Department of Orthopedics, The Affiliated Hospital of Traditional Chinese Medicine of Southwest Medical University, Luzhou, China.

**Keywords:** apoptosis, autophagy, bioinformatics, Duhuo, intervertebral disc degeneration, traditional Chinese medicine

## Abstract

Low back pain caused by intervertebral disc degeneration (IVDD), a common orthopedic disease in middle-aged and elderly populations, burdens patients’ quality of life and work capacity. IVDD is closely associated with nucleus pulposus cell apoptosis and autophagy, mediated by BCL2, CASP3, CASP8, and CASP9. Although Duhuo alleviates inflammation and delays IVDD, its molecular mechanism remains unclear. We used bioinformatics to identify IVDD-related apoptotic/autophagic pathways and Duhuo’s therapeutic mechanisms via network pharmacology. Bioinformatic analysis showed high expression of NLRP3, IL6, AKT1, and MMP9 in IVDD patients, with key cellular processes including senescence, apoptosis, autophagy, and ferroptosis, critical signaling pathways (HIF-1, FoxO, and PI3K-Akt), and main immune cells (T cells and Th17 cells). Network pharmacology identified Duhuo’s main active components (ammidin, isoimperatorin, β-sitosterol, O-acetylcolumbianetin, and angelol D) and 23 potential targets, among which BCL2, CASP3, CASP8, CASP9, ERR1, and PTGS1 are crucial. Enrichment analysis indicated Duhuo prevents IVDD by regulating apoptosis, autophagy, HIF-1, and PI3K-Akt pathways, and molecular docking confirmed β-sitosterol effectively binds key targets. This study identifies IVDD’s molecular basis and establishes a mechanism for targeted regulation of nucleus pulposus cell apoptosis/autophagy to prevent IVDD progression.

## 1. Introduction

Intervertebral disc degeneration (IVDD) is a common chronic disease in orthopedics that commonly occurs in middle-aged and elderly people. The low back pain (LBP) caused by it is the most important clinical symptom.^[1]^ LBP is one of the most common symptoms of spinal abnormalities and has become a serious public health problem worldwide, with a lifetime prevalence rate as high as 84%.^[2]^ The current clinical treatment strategies for alleviating IVDD mainly include conservative treatment and surgery.^[3]^ If conservative treatment fails, surgery is often considered for pain relief. However, these interventions are limited to symptom management rather than treatment of the underlying cause.^[4]^ Studies have found that the formation mechanism of IVDD is closely related to cellular aging, immune disorders, genetic factors, apoptosis, pyroptosis and increased release of inflammatory cytokines.^[5-9]^ Therefore, understanding the pathogenesis of IVDD is crucial to develop better solutions.

The normal intervertebral disc is a relatively closed environment, so the nucleus pulposus (NP) tissue lacks direct blood supply, and the nucleus pulposus cells (NPCs) live in a hypoxic and low-nutrient microenvironment.^[7,10]^ This special structure of the intervertebral disc makes NPCs very sensitive to changes in the microenvironment, especially to accumulated inflammatory factors.^[11]^ In recent years, studies have found that inflammatory response plays a crucial role in the pathogenesis and development of NP tissue degeneration, and its common feature is inflammatory cytokines such as tumor necrosis factor-α (TNF-α) and interleukin-1β (increased levels of IL-1β), etc., lead to an imbalance in the synthesis and degradation of extracellular matrix and promote the death of NPCs.^[2,12,13]^ Therefore, preventing or reducing the death of NPCs is considered to be the key to preventing IVDD and rebuilding intervertebral disc function.

Cell death can be divided into accidental cell death and regulated cell death.^[14]^ This paper will explore the mechanism of apoptosis and autophagy in IVDD.^[15-17]^ As one of the main ways of cell death, the abnormality of apoptosis is closely related to the occurrence and development of many diseases (including tumors).^[18]^ The causes of IVDD include oxidative stress, aging, nutritional deficiency, genetic susceptibility and mechanical load, etc.^[5]^ Studies have shown that the early stage of IVDD is accompanied by the infiltration of a large number of inflammatory factors, including IL-1β, TNF-α, IL-18, etc.^[6]^ These inflammatory factors, in addition to activating the pyroptosis mediated by the inflammasome NLRP3, leading to a decrease in the number and quality of NPCs, can also mediate cellular oxidative stress damage.^[5]^ Recent studies have found that the NLRP3 inflammasome is widely activated during IVDD, can mediate the production of various inflammatory cytokines, and can further participate in IVDD.^[19]^ Studies have found that autophagy and apoptosis regulated by ER stress (ERS) play an important role in the NPCs of IVDD. In both hypoxic and serum-deprived environments, LPS significantly activated ERS and significantly promoted autophagy and apoptosis. Overall, ERS influences the occurrence and progression of IVDD by regulating autophagy, apoptosis and other programmed death.^[20]^ In addition, inhibition of NF-κB pathway promoted LPS, induced autophagy in NPC and reduced apoptosis and inflammation.^[21]^ At the same time, we found that there are high expressions of key apoptosis proteins CASP and BCL2 in human intervertebral disc NPC, which indicates that apoptosis and autophagy may play an important role in IVDD, but the specific mechanism has not yet been elucidated.^[9]^

In traditional Chinese medicine, IVDD belongs to the category of “Bi syndrome” and “lumbar paralysis.” Duhuo, as the master of the prescriptions, is the main medicine in Duhuo Jisheng Decoction for removing paralysis. It is the main medicine for “all kinds of paralysis” and has the effect of dispelling wind and dampness, dispersing cold and relieving pain.^[22,23]^ Relevant clinical studies have also found that Duhuo Jisheng Decoction can directly reduce the levels of inflammatory markers and improve clinical symptoms by reducing inflammatory reactions.^[24]^ Lovage root has long been used to treat inflammation and arthritis. They are also used as analgesics and antipyretics for the treatment of headaches.^[25]^ Modern pharmacological studies have also found that the active ingredients of Duhuo have pharmacological effects such as delaying intervertebral disc degeneration, improving bone metabolism, inhibiting the activity of inflammatory mediators, and improving microcirculation, and are widely used in clinical practice.^[26,27]^ Studies have found that β-sitosterol,the main component of Solo, is a potential metabolic regulator in neurodegenerative diseases.^[28]^ Studies have found that β-sitosterol increases levels of antioxidant enzymes by activating the estrogen receptor/PI3-kinase pathway. It also regulates glutathione levels, indicating that it is an effective free radical scavenger.^[28]^ Furthermore, glucose oxidase-mediated oxidative stress and lipid peroxidation may be inhibited by β-sitosterol incorporation into cell membranes, suggesting a valuable role for this compound in neurodegenerative diseases including AD.^[29]^ In 1 study, β-sitosterol decreased the expression of pro-inflammatory markers such as IL-6, inducible nitric oxide (iNOS), TNF-α and cyclooxygenase-6 (COX-2), in Shows anti-inflammatory effects in BV2 cells when exposed to LPS. It also inhibits phosphorylation and degradation of nuclear factor κB (IκB) inhibitor, and inhibits nuclear factor κB (NF-κB) and extracellular signal-regulated kinase (ERK) phosphorylation, which regulates various cytokines in the inflammatory pathway.^[30]^ Although studies have found that β-sitosterol can improve inflammation, there is no related research in the intervertebral disc. As the key bioactive component of Duhuo, the effect of β-sitosterol is not limited to a single anti-inflammatory function. Instead, by binding to targets such as BCL2 and CASP3,^[31]^ it simultaneously inhibits excessive apoptosis, regulates autophagic balance, and is associated with the PI3K-Akt/HIF-1 signaling pathway.^[32]^ To date, no similar reports on this “bidirectional regulation” mechanism in the treatment of IVDD have been documented. Whether β-sitosterol can regulate inflammation It is not clear whether the inflammatory response of NPCs is affected by apoptosis, so elucidating the apoptotic molecular mechanism by which β-sitosterol regulates NPC death is of great significance.^[33,34]^ In order to discover the key genes regulating apoptosis, autophagy and inflammation in IVDD patients, we examined the GSE56081, GSE150408, and GSE124272 datasets in the Gene Expression Omnibus database and the Genecard database to collect hub genes of IVDD disease and apoptosis.^[35,36]^ We obtained 39 hub genes of IVDD that are closely related to apoptosis, autophagy and inflammation. In addition, based on the TCMSP traditional Chinese medicine database, we conducted an in-depth analysis of the molecular mechanism of Duhuo in treating IVDD. In order to further verify our prediction, we carried out molecular docking verification, and finally we proved that the regulation of NPC apoptosis and autophagy is the key to the treatment of IVDD, and Duhuo can effectively act on this signaling pathway to improve IVDD. We believe that this study will provide a solid theoretical basis for the treatment of IVDD and promote the clinical application of traditional Chinese medicine.

## 2. Materials and methods

### 2.1. Original data collection and differential gene analysis

Sequencing datasets for IVDD were collected from the NCBI Gene Expression Omnibus public database (GEO). The datasets we use include GSE56081, GSE124272, GSE150408.^[37]^ Convert the 3 chip matrix data into gene name data, and then perform data fusion and batch removal processing to obtain the original matrix data after removing batch effects. Based on the original data of the matrix, use the “limma” tool to screen differential genes, obtain differentially regulated genes, and draw a differential volcano map.^[38]^ In addition, the setting parameters are: difference fold (log_2_FC) > 1.2 and the false discovery rate < 0.05, distance calculation method: euclidean, clustering method: complete, and draw a differential gene heat map.

### 2.2. Weighted correlation network analysis

Weighted gene co-expression network analysis (WGCNA) software package of Sanger-box 3.0 was used to construct the gene co-expression network.^[39]^ According to the clustering tree, the top 50% genes with the smallest MAD were eliminated. Calculate the correlation coefficient between each gene pair and construct a similarity matrix. To ensure the construction of the scale-free network, an appropriate soft threshold is chosen to transform the similarity matrix into an adjacency matrix. Subsequently, a topological overlap matrix was created to measure the average network connectivity of each gene. Genes with similar expression profiles were grouped into different modules using a dynamic tree cut method based on the relevant parameters of the block-dimensional module functions. Each module is depicted with a different color, where genes in gray modules represent genes that cannot be assigned to any module. The gene expression profile of each module is composed of the first principal component called the module eigengene (ME). MEs are used to evaluate the association between modules and phenotypes. Determine the module with the highest absolute value of the correlation coefficient as the key module for further analysis. Module membership (MM) is the correlation coefficient between the expression value of a gene and the ME of a module, indicating the correlation between the gene and the module. Gene significance (GS) is the correlation coefficient between the expression value of a gene and a phenotype, representing the correlation between the gene and the phenotype. Set the MM threshold to 0.8, the GS threshold to 0.1, and the weight threshold to 0.1 to extract hub genes. The thresholds MM (modularity) > 0.8 and GS (genetic significance) > 0.1 were selected based on the following rationale: As established in WGCNA studies,^[40]^ these thresholds effectively filter out core genes with high correlations (*r* > 0.8) between modular eigenvectors and disease phenotypes, thereby excluding low-association “noise genes”; preliminary experimental results demonstrate that when MM > 0.8, intra-module gene co-expression consistency exceeds 90%, while when GS > 0.1, the correlation between genes and IVDD phenotypes achieves statistically significant levels (*P* < .01), ensuring the selected hub genes possess biological significance.

### 2.3. Hub gene screening

Using “intervertebral disc degeneration” as the keyword, search results in Genecards (https://www.genecards.org/), Comparative Toxicogenomics Database (https://ctdbase.org/), DisGeNET (https://www.disgenet.org/) disease database to obtain target proteins of the disease.^[41]^ Then, the collected disease target data and differentially regulated genes are intersected to obtain hub gene set A. Intersect the collected disease target data with module genes to obtain hub gene set B. By merging the 2 hub gene sets A and B, the disease regulation hub gene set C of IVDD is obtained. Using the S TRING database (http://string-db.org/) to conduct protein–protein interaction network analysis on the hub gene set C of IVDD, this database is limited to “Homo sapiens,” and the confidence value is >0.4 ^[42]^ In addition, we used the CytoHubba algorithm of the Cytoscape 3.9.1 software to screen the obtained hub gene set C to obtain the top 20 hub genesto construct the action network. Finally, based on the hub genes that effectively regulate IVDD, we selected the top 10 genes that play a key role in the network to compare and calculate the disease group and the normal group, so as to initially verify the differential expression of these genes.

### 2.4. Enrichment analysis of hub genes

Based on hub gene set A, KEGG enrichment pathway analysis was performed. Based on hub gene set B, KEGG enrichment pathway analysis was performed. Finally, GO enrichment analysis was performed based on gene set C. The KEGG signaling pathway analysis is displayed in a histogram, and the GO enrichment analysis is displayed in a circle diagram. In addition, based on the results of the comparison of the top 10 differential genes, we selected statistically significant genes (*P* < .05) to conduct analysis of the key pathways of single genes in diseases. The results are shown in the Gene Set Enrichment Analysis graph.

### 2.5. Molecular mechanism of Duhuo in treating IVDD

Use the analysis platform of Traditional Chinese Medicine Systems Pharmacology Database (TCMSP, https://tcmspw.com/tcmsp.php) to collect the active ingredients and targets of Duhuo. According to the criteria of oral bioavailability ≥ 30% and drug similarity (DL) ≥ 0.18,^[43]^ as well as absorption, distribution, metabolism and excretion (ADME) procedures, the active ingredients of the active ingredients were collected. Based on the effective substance, we collect the action targets of each effective active ingredient, and finally fuse all the targets to obtain the unique action target data set D. The data set E of the potential therapeutic target is obtained by intersecting the original data set D of the independent living drug target with the original data of IVDD. The enrichment analysis of the intersection therapeutic targets (data set E) was performed to obtain the KEGG signaling pathway expression results. In addition, the CytoHubba algorithm of Cytoscape 3.9.1 software was used again to screen the obtained hub gene set E to obtain key targets for treatment, and to compare the disease and normal group data in the original data to verify the differences in these targets Condition. Finally, use Cytoscape 3.9.1 to construct a regulatory network diagram of “Indigo – Active Ingredients-Key Gene – Signaling Pathway – IVDD”.

### 2.6. Molecular docking verification

Through the previous analysis, we have initially obtained the active ingredients and key targets of Duhuo’s treatment of IVDD, but whether this prediction is credible requires further verification. To this end, molecular docking calculations based on bioinformatics calculations can initially prove the binding ability of the active ingredients to the target protein, thereby proving the drug’s mechanism of action. Therefore, we followed the following steps for molecular docking verification.^[44,45]^

(1) Ligand processing: obtain the 3-dimensional structure of the proposed docking target in mol2 format from the Pubchem database, use AutodockTools 1.5.6 to open the small ligand molecule, hydrogenate, charge, detect the ligand root, search and define rotatable bonds, Then save it as a pdbqt file.
(2) Receptor processing: download the core 3-dimensional structure of the target protein from the RCSB protein database (www.rcsb.org/) as a docking protein. Add all hydrogen atoms in AutodockTools 1.5.6 to open, calculate Gasteiger charge, combine nonpolar hydrogen, define as acceptor, and save as pdbqt file.^[46]^
(3) Docking parameter setting: determine the coordinates and box size of the Vina molecular docking, set the exhaustive parameters to 15, and take the default values for other parameters.
(4) Operation and output. Autodockvina 1.1.2 was used for semi-flexible docking, and the conformation with the best affinity was selected as the final docking conformation.

## 3. Results

### 3.1. Differential gene analysis results

**Figure 1.**
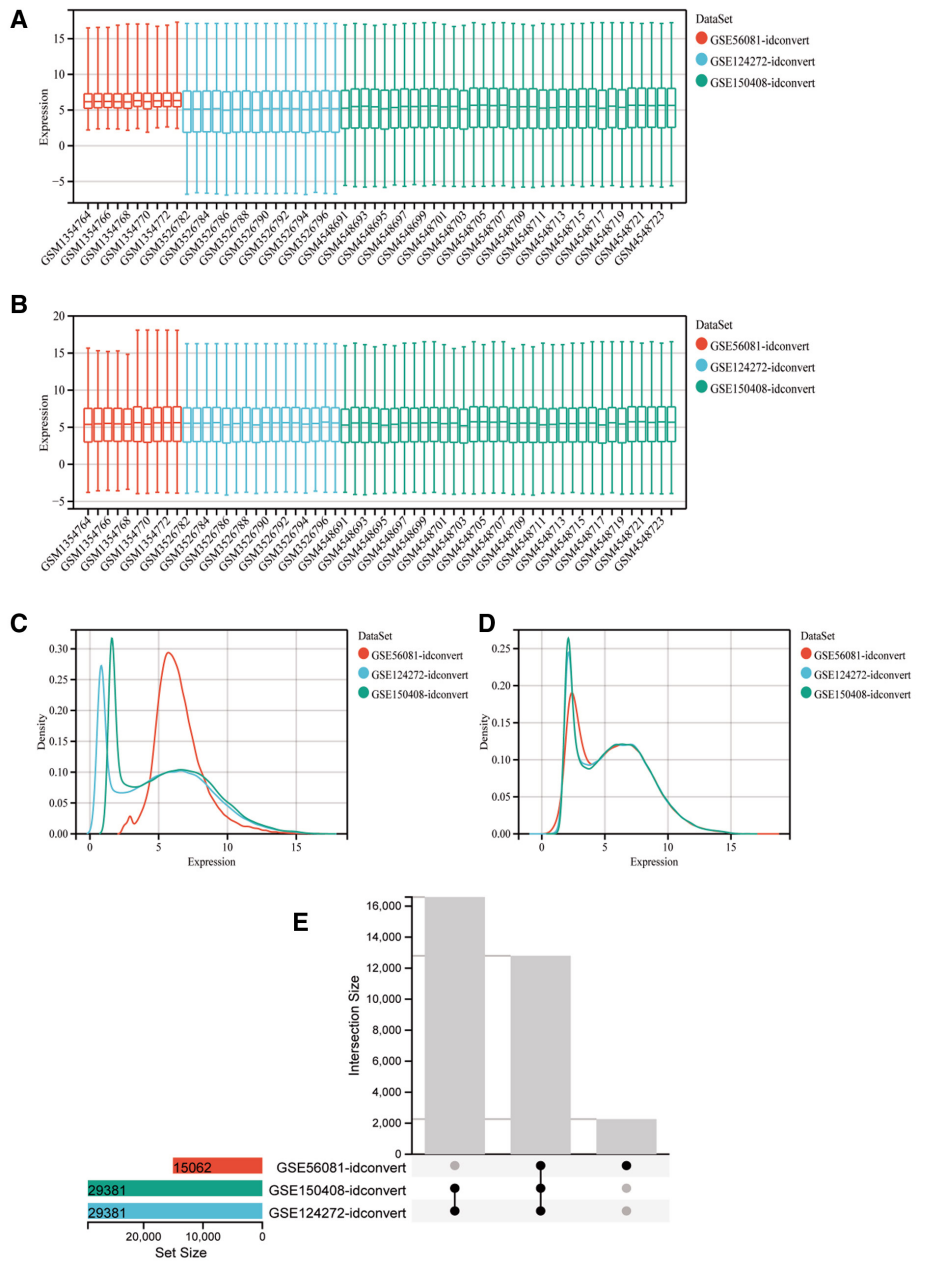
De-batch processing effect: (A) the graph shows that before removing the batch effect, the samples in each dataset are clustered together, which suggests the presence of a batch effect. (B) After removing the batch effect, the distribution of data between the datasets tends to be consistent, with similar means and variances. (C) The density plot shows that before removing the batch effect, the distribution of samples in each dataset varies greatly, which suggests the presence of a batch effect. (D) After the batch effect is eliminated, the data distributions between the data sets converge, with similar means and variances. (E) The plot shows the intersection of the data sets.

**Figure 2.**
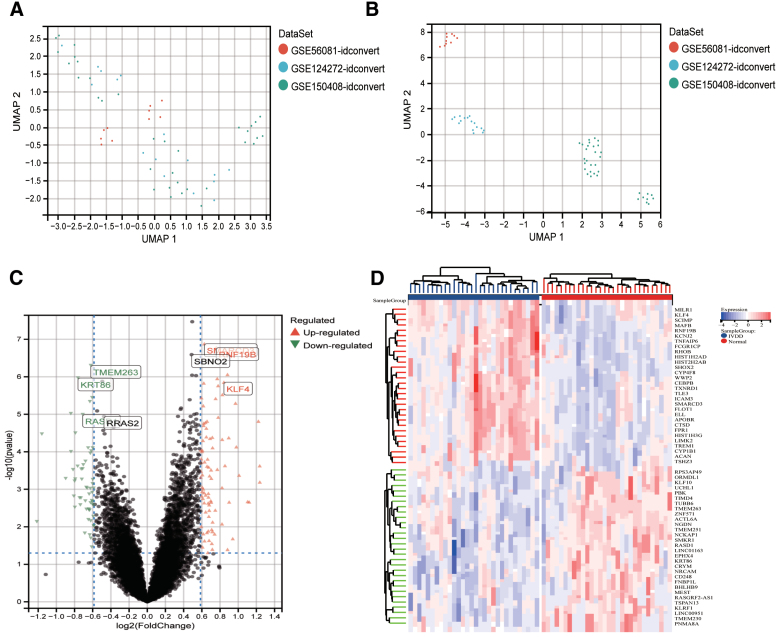
(A) Before correction, samples from different datasets show obvious clustering, indicating batch effects. (B) After correction, the IVDD group and the normal group show clear clustering, and differences between datasets are eliminated, confirming the validity of data integration. (C) Differential volcano plot showing 1558 significant differential genes out of 12,799 total genes, including 878 up-regulated genes and 680 down-regulated genes. (D) Differential heatmap demonstrating the level of difference between the top-ranked differential genes in the normal group and the IVDD disease group. IVDD = intervertebral disc degeneration.

Download the GSE56081, GSE124272, and GSE150408 datasets from the GEO database. Among them, the post-treatment samples were removed from the GSE 150408 data, and finally the 60 samples included in this data included 30 IVDD samples and 30 normal samples. GSE56081 includes 5 I VDD samples and 5normal samples, GSE 124272 includes 8 IVDD samples and 8 normal samples, and GSE150408 includes 17 IVDD samples and 17 normal samples (Tables 1 and 2). Matrix expression data containing gene names were obtained after gene ID conversion, data fusion, and debatch processing (Fig. 1, Table S1, Supplemental Digital Content). To verify the effect of batch effect correction, we performed principal component analysis on the corrected and pre-data (Fig. 2A, B). The results showed that the corrected samples clustered by disease status (rather than data set source), indicating that the quality of data integration was reliable. Based on the matrix expression data, we performed differential expression analysis using the “limma” tool. The volcano plot of differences between IVDD samples and normal control samples showed 1286significantly different genes (DEGs) out of 12,799 total genes, including 792 up-regulated genes and 494 down-regulated genes (Fig. 2C, Table S2, Supplemental Digital Content). Further use the difference heat map to display the difference level of the top-ranked difference genes between the normal group and the IVDD disease group (Fig. 2D).

**Table 1:** Descriptive statistics.

Data number	Platform information	IVDD group	Control group	Species
GSE56081	GPL15314	5	5	Homo sapiens
GSE150408	GPL21185	17	17	Homo sapiens
GSE153761	GPL22120	3	3	Homo sapiens

IVDD = intervertebral disc degeneration.

**Table 2:** Active ingredients of Duhuo.

Mol ID	Molecule name	OB (%)	DL
MOL001941	Ammidin	34.55	0.22
MOL001942	Isoimperatorin	45.46	0.23
MOL000358	β-sitosterol	36.91	0.75
MOL003608	O-acetylcolumbianetin	60.04	0.26
MOL004777	Angelol D	34.85	0.34
MOL004778	[(1R,2R)-2,3-dihydroxy-1-(7-methoxy-2-oxochromen-6-yl)-3-methylbutyl] (Z)-2-methylbut-2-enoate	46.03	0.34
MOL004780	Angelicone	30.99	0.19
MOL004782	[(1R,2R)-2,3-dihydroxy-1-(7-methoxy-2-oxochromen-6-yl)-3-methylbutyl] 3-methylbutanoate	45.19	0.34
MOL004792	Nodakenin	57.12	0.69

DL = drug similarity.

### 3.2. WGCNA results

**Figure 3.**
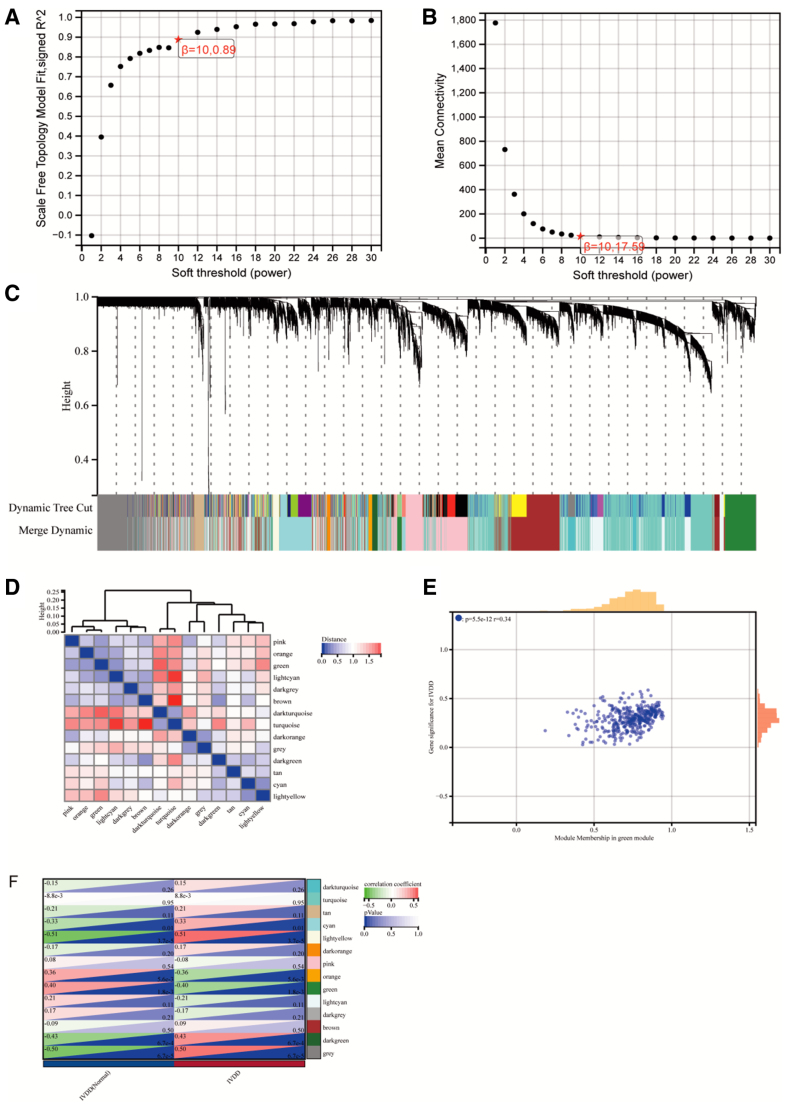
(A, B) When the soft threshold power was set to 10, the scale independence reached 0.89 and the average connectivity value was 17.59. When the cut height was set to 0.25 and the minimum module size was set to 30. (C) 14 different co-expression modules were obtained by dynamic tree cutting. (D) Individual modules were subjected to the modular eigenvector clustering analysis showing that the turquoise vs brown, the and turquoise with lightcyan modules distance was the largest. (E) Correlation analysis between MM and GS showed that these genes were highly correlated with both modules and phenotypes (*r* = 0.34, *P* = 5.5e−12,). (F) The cyan module was positively correlated with IVDD (correlation coefficient = 0.33, *P* = .01), and the remaining modules were not statistically significantly associated with IVDD. GS = gene significance, IVDD = intervertebral disc degeneration, MM = module membership.

About 12,799 samples were extracted from the 3 data sets and used to construct a weighted gene co-expression network. When the soft threshold power was set to 10, the scale independence reached 0.89 and the average connection value was 17.59 (Fig. 3A, B). When the cutting height was set to 0.25 and the minimum module size was set to 30, 14 different co-expression modules were obtained through dynamic tree cutting (Fig. 3C). Cluster analysis of module feature vectors for each module shows that turquoise and brown, as well as turquoise and lightcyan modules have the largest Distance (Fig. 3D). Correlation analysis between MM and GS showed that these genes were highly correlated with both modules and phenotypes (*r* = 0.34, *P* = 5.5e − 12, Fig. 3E). Then, the correlation analysis between each module and clinical characteristics was performed. The cyan module is positively correlated with IVDD (correlation coefficient = 0. 33, *P* = 0. 01), and the other modules have no significant statistical significance with IVDD (Fig. 3F). In addition, based on the classification of each module, a total of 1540 module genes were finally extracted (Table S4, Supplemental Digital Content).

### 3.3. Hub gene

**Figure 4.**
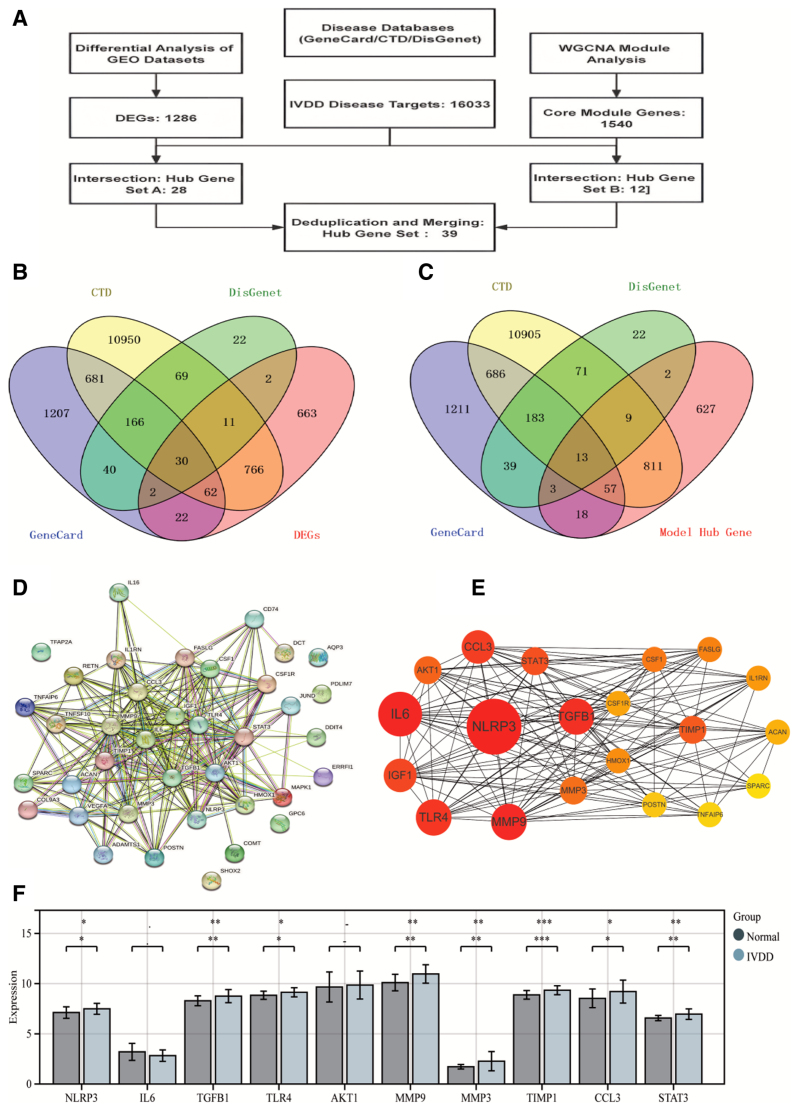
(A) Flowchart of hub gene screening. (B) Taking the intersection of differential genes with disease data genes, we obtain 30 intersecting gene sets A. (C) Taking the intersection of modular genes with disease data genes, we obtain 13 intersecting gene sets B (D) Constructing a PPI network using STING tool shows that these 39 intersecting genes interact with each other. (E) After calculating the top 20 important regulatory hub genes we obtain the top 20 important regulatory hub genes. (F) Differential Statistical analysis Expression analysis showed that the hub genes NLRP3, IL6, TGFB1, TLR4, AKT1, MMP9, MMP3, TIMP1, and SATA3 may play important roles in IVDD. CTD = C-terminal domain, DEG = differential gene, GEO = Gene Expression Omnibus, IVDD = intervertebral disc degeneration, PPI = protein–protein interaction, STING = Stimulator of Interferon Genes, WGCNA = weighted gene co-expression network analysis.

The analysis of Duhuo treatment for IVDD was summarized from 4 databases (Fig. 4A). By downloading the disease database, we obtained the disease-related targets of IVD. The GeneCard database contained 2210 disease targets, the C-terminal domain database had 13,156 disease targets, and the DisGenet database included 667 disease targets (Fig. 4B, C, Tables S3 and S4, Supplemental Digital Content) construction of the protein–protein interaction network using the STRING tool revealed these 39 intersection gene interactions (Table S5, Supplemental Digital Content, Fig. 4D). Furthermore, after calculations, we identified the top 20 important regulatory hub genes (Fig. 4E). Differential statistical analysis and expression analysis indicated that among the hub genes, NLPR3, IL6, TGFB1, TLR4, AKT1, MMP9, MMP3, TIMP1, and STAT3, etc, may play significant roles in IVDD (Fig. 4F, Table S6, Supplemental Digital Content).

### 3.4. Enrichment analysis

**Figure 5.**
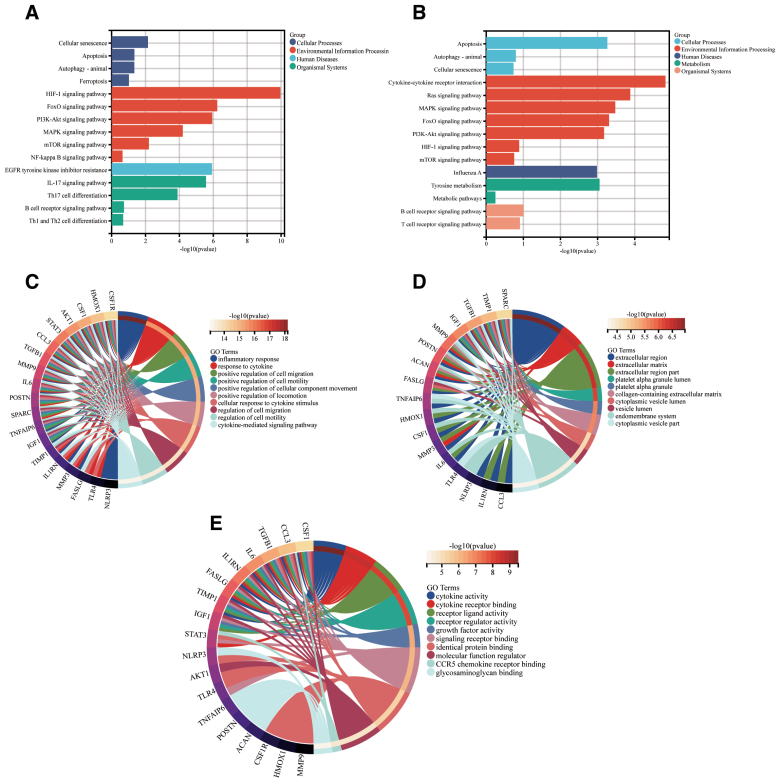
(A) Based on gene set (A) we found the major KEGG signaling pathway expression in the process of IVDD. (B) Based on gene set (B) we found the major KEGG signaling pathway expression in the process of IVDD. (C) Biological Process enrichment result of the 20 hub genes based on gene set C. (D) Cellular component enrichment result of the first 20 hub genes based on gene set C. (E) Molecular Function enrichment result of the first 20 hub genes based on gene set C. IVDD = intervertebral disc degeneration, KEGG = Kyoto encyclopedia of genes and genomes.

**Figure 6.**
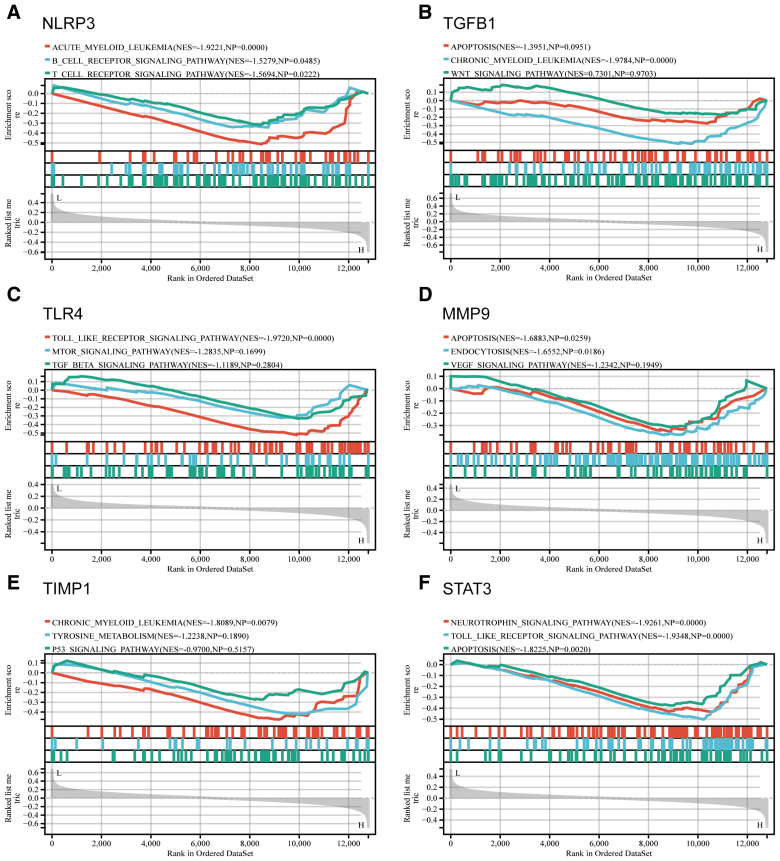
(A) Signaling pathways mainly involved in intervertebral disc degeneration by NLRP3 targets. (B) Signaling pathways mainly involved in intervertebral disc degeneration by TGFB1 targets. (C) Signaling pathways mainly involved in intervertebral disc degeneration by TLR4 targets. (D) Signaling pathways mainly involved in intervertebral disc degeneration by MMP9 targets. (E) Signaling pathways mainly involved in intervertebral disc degeneration by TIMP1 targets. (F) Signaling pathways mainly involved in intervertebral disc degeneration by SATA3 targets.

To further analyze how hub regulatory genes are involved in IVDD, we performed KEGG signaling pathway analysis. The results show that based on gene set A, we found that the main cellular processes in the IVDD process include apoptosis, cellular senescence, and autophagy. The main signaling pathways involve H IF-1 signaling pathway, PI3K-Akt signaling pathway, FoxO signaling pathway, and MAPK. Signaling pathway, NF-κ B signaling pathway, mTOR signaling pathway. In addition, we also found that the regulation of these genes is associated with immune cell responses, mainly involving Th 17 cell differentiation, B cell receptor signaling pathway. Based on gene set B, we found that the main cellular processes in the IVDD process also include cell senescence, apoptosis, and autophagy. The main signaling pathways involve HIF-1 signaling pathway, PI3K-Akt signaling pathway, FoxO signaling pathway, and Ras signaling. Pathway signaling pathway (Fig. 5A, B, Tables S7 and S8, Supplemental Digita Content). Finally, the GO enrichment results based on the top 20 hub genes of gene set C show: biological process mainly includes inflammatory response, response to cytokine, positive regulation of cell migration, positive regulation of cell motility, positive regulation of cellular component movement, positive regulation of locomotion. Cellular component is mainly in extracellular region, extracellular matrix, extracellular region part, platelet alpha granule lumen, platelet alpha granule. Molecular Function mostly involves cytokine activity, cytokine receptor binding, receptor ligand activity, receptor regulator activity, growth factor activity (Fig. 5C, D, E, Table S9, Supplemental Digital Content). In addition, based on the hub genes with statistically significant differences in NLRP3, T GFB1, T LR4, MMP9, TIMP1, and SATA3, we performed a pathway enrichment analysis of single genes in the samples. The results show that: NLRP3 targets are mainly involved in the signaling pathways of intervertebral disc degeneration: ACUTE_MYELOID_LEUKEMIA,B_CELL_RECEPTOR_SIGNALING_PATHWAY, T_CELL_RECEPTOR_SIGNALING_PATHWAY (Fig. 6A). The signaling pathways mainly involved in TGFB1 targets in intervertebral disc degeneration are: APOPTOSI, CHRONIC_MYELOID_LEUKEMIA, WNT_SIGNALING_PATHWAY (Fig. 6 B). The signaling pathways mainly involved in T LR4 targets in intervertebral disc degeneration: TOLL_LIKE_RECEPTOR_SIGNALING_PATHWAY, MTOR_SIGNALING_PATHWAY, TGF_BETA_SIGNALING_PATHWAY (Fig. 6C). The signaling pathways mainly involved in MMP9 targets in intervertebral disc degeneration are: APOPTOSI, ENDOCYTOSIS, VEGF_SIGNALING_PATHWAY (Fig. 6D). The signaling pathways mainly involved in T IMP1 targets in intervertebral disc degeneration are: CHRONIC_MYELOID_LEUKEMIA, TYROSINE_METABOLISM, P53_SIGNALING_PATHWAY (Fig. 6E). The signaling pathways mainly involved in SATA3 targets in intervertebral disc degeneration are: NEUROTROPHIN_SIGNALING_PATHWAY, TOLL_LIKE_RECEPTOR_SIGNALING_PATHWAY, APOPTOSIS (Fig. 6F). In summary, we found that the process of IVDD is mainly closely related to apoptosis.

### 3.5. Molecular mechanism of Duhuo in treating IVDD

**Figure 7.**
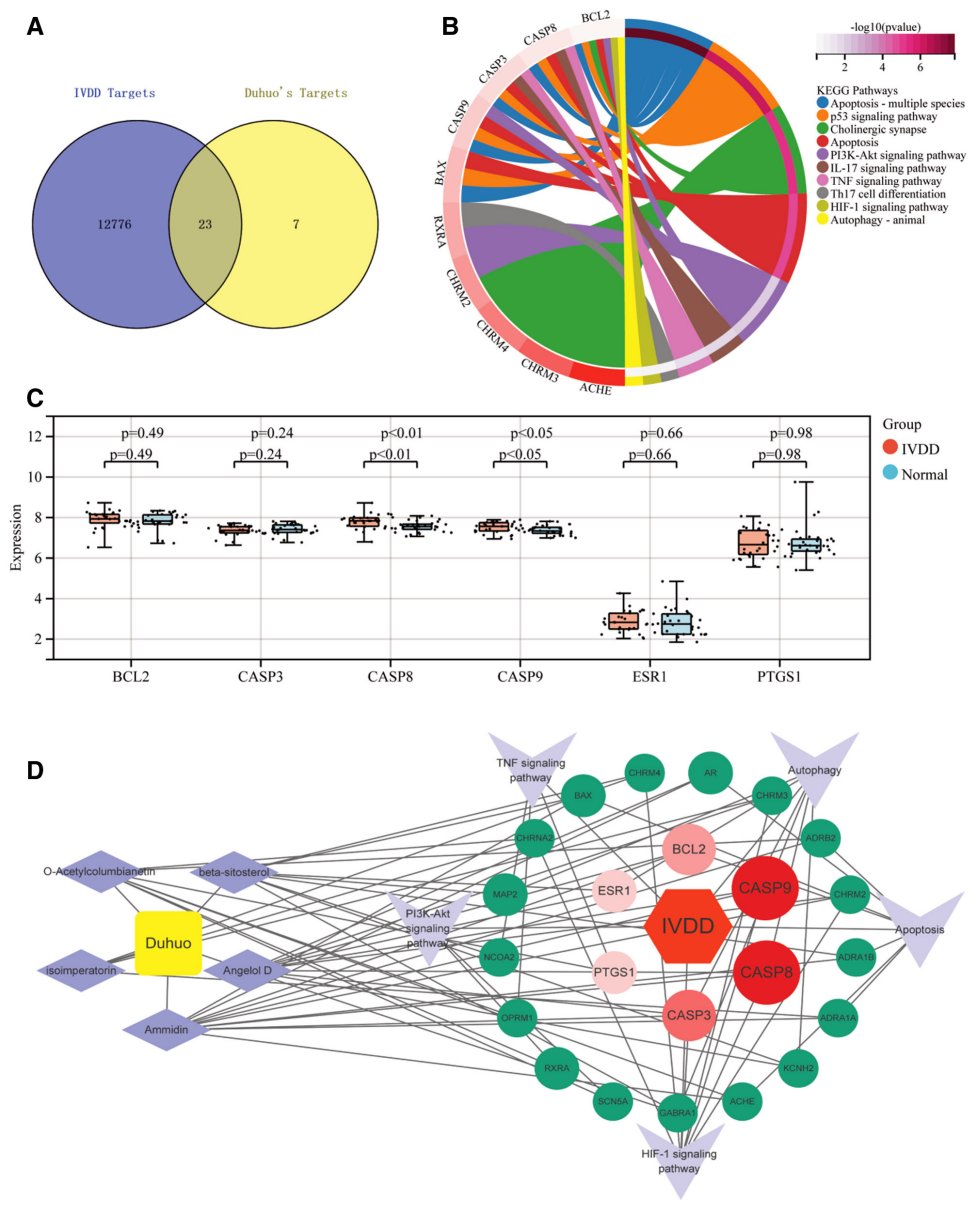
(A) Drug targets of action and original matrix effective targets were taken to intersect to obtain 23 potential therapeutic targets. (B) KEGG signaling pathways involved in the treatment of IVDD by Duhuo. (C) Difference-in-difference analysis showed that BCL2, CASP3, CASP8, CASP9, ESR1, and PTGS1 played a key role in the occurrence and treatment of IVDD, and CASP8 and CASP9 had a significant statistical significance (*P* < .05). (D) Diagram of the active ingredients, key signaling pathways and effective targets of action regulation network involved in IVDD treatment by Duhuo. IVDD = intervertebral disc degeneration, KEGG = Kyoto encyclopedia of genes and genomes.

**Figure 8.**
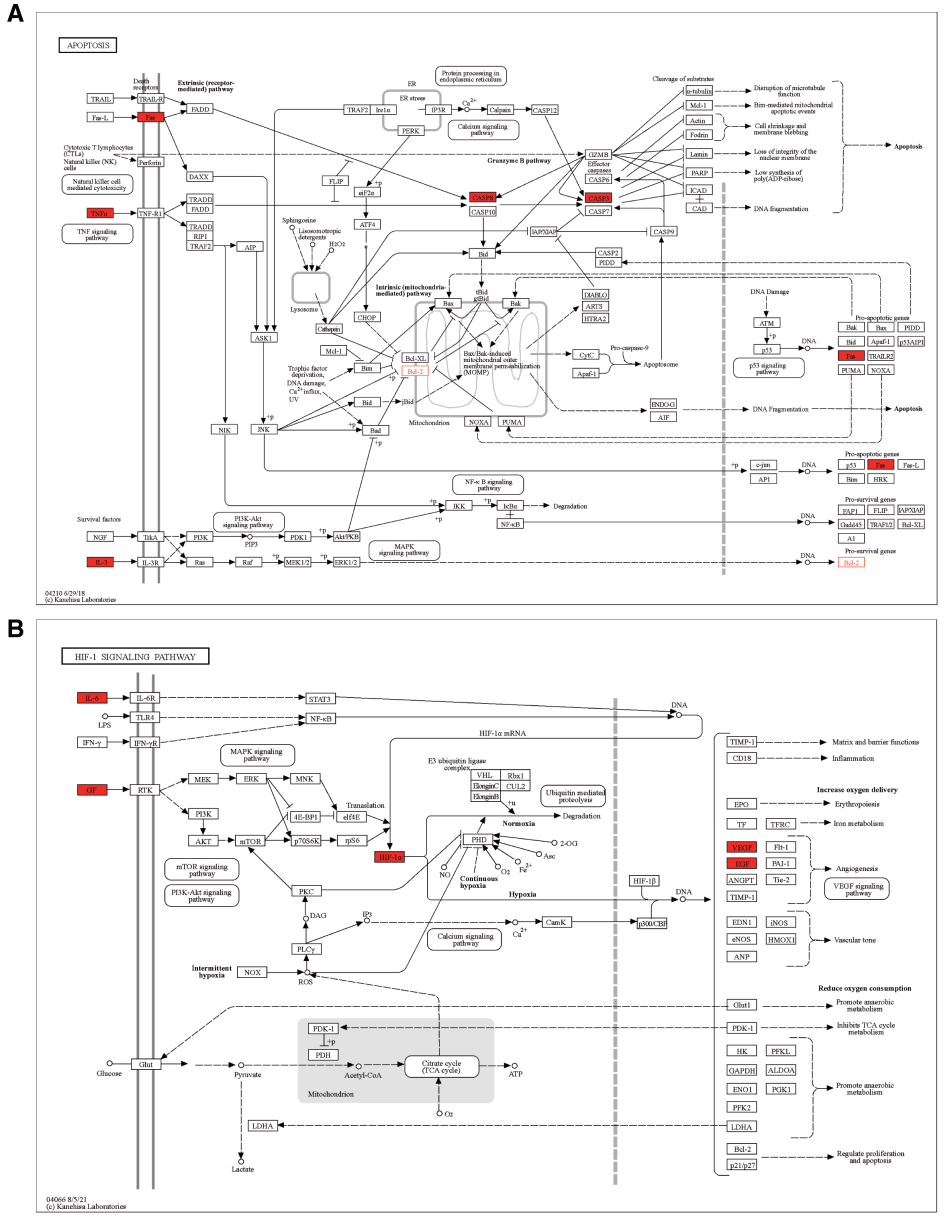
The process of apoptosis, HIF-1 signaling pathway, which involves BCL2, CASP3, CASP8, CASP9 and other targets are consistent with our analysis.

Through TCMSP screening, we obtained 9 active ingredients of solva, among which ammidin, isoimperatorin, β-sitosterol, O-acetylcolumbianetin and Angelol D may play the main therapeutic role (Table 2). Based on the active ingredients, we finally obtained 30 unique and effective targets. The intersection of drug action targets and original matrix effective targets resulted in 23 potential therapeutic targets (Fig. 7A, Table S10, Supplemental Digital Content). Based on the KEGG enrichment analysis of 23 targets, the signaling pathways involved in the treatment of IVDD by Duhuo mainly include apoptosis, PI3K-Akt signaling pathway, IL-17 signaling pathway, HIF-1 signaling pathway, p53 signaling pathway, etc (Fig. 7B). Combined with the disease enrichment analysis of IVDD, we found that apoptosis and autophagy may play important roles in the occurrence and treatment of diseases. We conducted differential analysis and verification on the key proteins of apoptosis, autophagy and HIF-1 again, and the results showed that B CL2, C ASP3, CASP8, C ASP9, ESR1, and P TGS1 played key roles in the occurrence and treatment of IVDD. Among them, CASP8 and C ASP9 were statistically significant (*P* < .05; Fig. 7C, Table S11, Supplemental Digital Content). Finally, we used the network regulation map to demonstrate the active components, key signaling pathways and effective targets involved in the treatment of IVD by lovage (Fig. 7D). In addition, we used the K EGG database to download the signaling pathway diagrams of apoptosis and H IF-1. This figure clearly shows the process of apoptosis and HIF-1 signaling pathway, and the targets involved in BCL2, CASP3, CASP8, CASP9 are consistent with our analysis results (Fig. 8).

### 3.6. Molecular docking results

**Figure 9.**
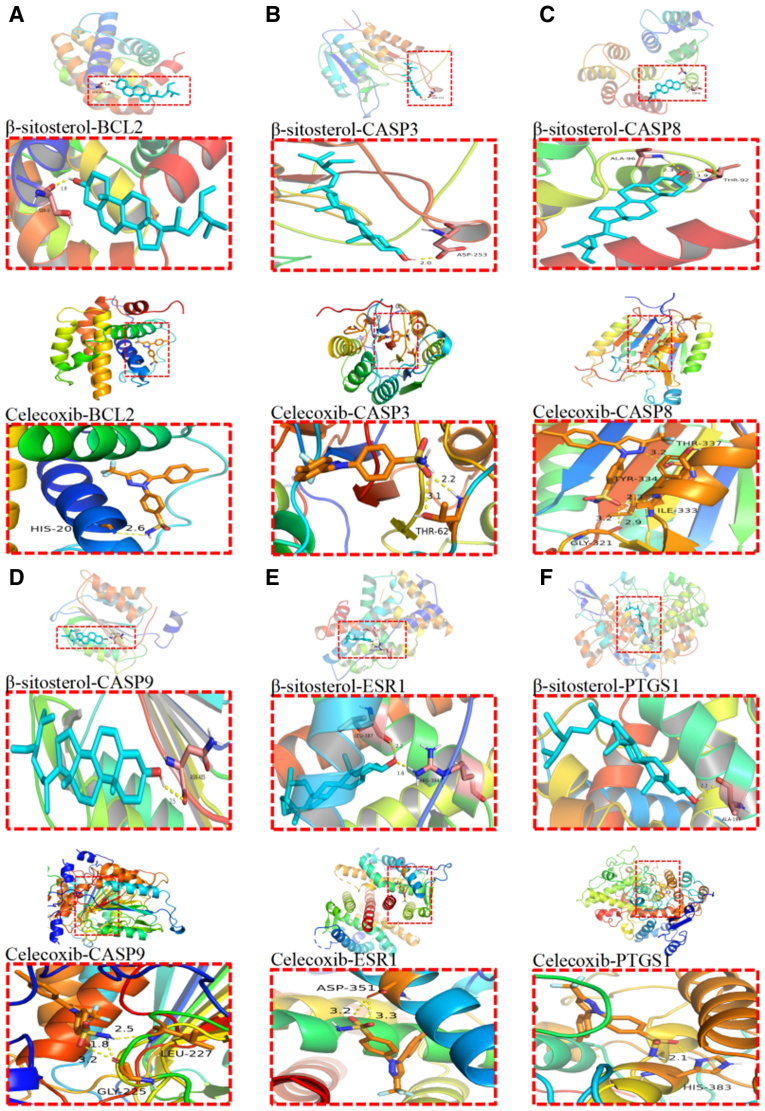
Results of molecular docking of solo active sufficient with key targets: (A) results of β-sitosterol and Celecoxib vs BCL2. (B) Results of β-sitosterol and Celecoxib vs CASP3. (C) Results of β-sitosterol and Celecoxib vs CASP8. (D) Results of β-sitosterol and Celecoxib vs CASP9. (E) Results of β-sitosterol and Celecoxib vs ESR1. (F) Results of β-sitosterol and Celecoxib vs PTGS1.

To further elucidate the mechanism of action of Duhuo in treating IVDD, we analyzed and docked the key components of Duhuo, including β-sitosterol, with its core targets BCL2, CASP3, CASP8, CASP9, ESR1, and PTGS1. We also introduced celecoxib as a positive control group (Fig. 9, Table S12, Supplemental Digital Content). The results indicated that β-sitosterol had higher docking scores with BCL2, CASP3, CASP8, CASP9, ESR1, and PTGS1 than celecoxib, and the binding energies of all ligands were below −5 kcal/mol suggest that β-sitosterol, as one of the main components of Duhuo, may play a crucial role in the treatment of IVDD.

## 4. Discussion

According to statistics, more than 84% of people around the world experience LPB, and IVDD is a major pathological factor for LBP, but its pathological mechanism has not been fully elucidated.^[47]^ At present, most opinions believe that NPCs death and immune inflammation are closely related to various pathological processes of IVDD.^[5]^ Specifically, IL-1β and TNF-a mediated inflammation of NPCs are closely related to NPCs apoptosis and autophagy IVDD mediated by CASP.^[19]^ Although the mechanisms of inflammatory expression and apoptosis pathways in IVDD have been extensively studied, it’s specific molecular mechanisms need to be further improved. Therefore, we will discover the mechanism of apoptosis and inflammation in IVDD in this study, and provide guidance for the clinical diagnosis and treatment of IVDD. In addition, traditional Chinese medicine, as a traditional medicine in China, has good effects in improving LBP in patients with IVDD. Among them, Duhuo has a significant effect in regulating intervertebral disc inflammation, but the specific molecular mechanism is unclear. In this study, we further clarified the molecular mechanism of apoptosis and autophagy that Duhuo regulates.^[48]^

The core pathological feature of IVDD lies in the functional loss and death of NPCs, where apoptosis as the primary form of NPC death plays a key role in disease progression due to regulatory imbalance. Through WGCNA screening, this study identified 39 hub genes showing strong correlations with IVDD phenotype (GS > 0.1, MM > 0.8) including key molecules in apoptosis pathways such as BCL2 and CASP3/8/9. This indicates that the apoptosis pathway does not act alone in pathogenesis but forms an interdependent regulatory network with autophagy and oxidative stress pathways – aligning with previous conclusions that “IVDD is a multi-pathway coordinated disorder.”^[6]^ Further molecular docking validation revealed that the core component β-glycosterol of Duhuo could stably bind to BCL2 and CASP3/8/9 (binding energy < −5 kJ·mol^−1^), suggesting its potential to disrupt the vicious cycle of “apoptosis excess-matrix degradation” in NPCs through direct regulation of apoptosis pathway molecules, rather than merely influencing downstream inflammatory factor expression.

Through bioinformatics, we found that the process of IVDD mainly involves abnormal expression of signaling pathways such as apoptosis, cell senescence, autophagy, PI3K-Akt signaling pathway, FoxO signaling pathway, and NF-κB signaling pathway. The main inflammatory factors and regulatory targets are NLRP3, AKT1, IL6, IL-1β, TNF-a, B CL2, CASP3, CASP8, CASP9, ESR1, PTGS1. Studies have found that apoptosis contributes to the development of IVDD, which can be activated by various types of stimuli through death receptor, endoplasmic reticulum and mitochondrial pathways.^[15,49]^ Mitochondrial dysfunction caused by oxidative stress, including the reduction of Bcl-2 and the release of Bax, triggers the activation of caspase family (CASP3, CASP8), leading to apoptosis.^[50]^ In this study, we predicted through molecular docking that β-sitosterol can inhibit the IL-1β, TNF-a, CASP3, CASP8 in the degenerated intervertebral disc, and increase Bcl-2 at the same time, which will effectively reduce the NPCs. The expression of inflammatory response and apoptosis signal improves intervertebral disc degeneration.

The core active component of Angelica pubescens, β-sitosterol, precisely targets the pathological pathway of the “oxidative stress-inflammation-apoptosis” vicious cycle in IVDD due to its antioxidant and anti-inflammatory properties. In IVDD, the hypoxic microenvironment of the NP induces massive ROS production. The accumulation of ROS exacerbates mitochondrial damage (promoting BCL2 downregulation and cytochrome C release) and activates the NF-κB inflammatory pathway.^[51]^ β-Sitosterol activates the estrogen receptor (ER)/PI3K pathway to upregulate antioxidant molecules such as superoxide dismutase (SOD) and glutathione (GSH),^[29]^ directly scavenging excess ROS within NPCs and blocking the “ROS-mitochondrial damage-poptosis” cascade. Simultaneously, it embeds into the cell membrane to maintain lipid structural stability, reducing ROS-mediated lipid peroxidation and protecting NPCs from oxidative stress damage. NLRP3 inflammasome activation leads to sustained release of inflammatory mediators like IL-1β and IL-6. β-Sitosterol blocks NF-κB nuclear translocation by inhibiting IκB phosphorylation and degradation,^[30]^ while simultaneously downregulating ERK/p38 MAPK pathway activity.^[27,52]^ This dual inhibition of cytokine transcription and release not only reduces CASP8-mediated apoptosis activation but also alleviates inflammation-induced suppression of ECM synthesis (e.g., by reducing MMP9-mediated collagen degradation, thereby simultaneously improving NPC survival and ECM homeostasis.

In addition, in addition to apoptosis, we also found that autophagy, PI3K-Akt signaling pathway, FoxO signaling pathway, NF-κB signaling pathway and other signaling pathways are expressed in.^[5]^ In addition, the rate of disc aging is also associated with high levels of reactive oxygen species (ROS) production. ROS are unavoidable during cellular aerobic metabolism. When stressed, ROS accumulate in cells, causing oxidative damage and cellular damage to proteins, DNA, and lipids, leading to an imbalance in autophagy.^[53]^ Generally speaking, inflammatory tissue is associated with increased levels of ROS produced by immune cells. Once cellular pro-oxidative and antioxidant systems fail, excessive inflammation will occur and ROS production will increase.^[54]^ IVDD is associated with local increases in IL-1β and TNF-a, which may be because IL-1β can induce apoptosis through mitochondrial dysfunction and endoplasmic reticulum stress.^[54]^ Elevated ROS activated multiple signaling pathways in intervertebral cells during oxidative stress, including PI3K-Akt signaling pathway, FoxO signaling pathway, NF-κB signaling pathway. NF-κB is a transcription factor that regulates genes encoding pro-inflammatory cytokines and is a potential therapeutic target for various inflammatory diseases.^[55]^ It has been shown that inhibition of the NF-κB pathway promotes LPS, induced autophagy in NPCs and reduces apoptosis and inflammation. Meanwhile, autophagy triggered by NF-κB inhibition plays a protective role against apoptosis and inflammation.^[21]^ Meanwhile, the research team previously found that the SDF-1/CXCR4 axis promotes human degenerative NPC cell apoptosis through the NF-κB pathway.^[9]^ The latest study of the research group found that DHJSD inhibited the pyroptosis of NPCs through the SDF-1/CXCR 4-NF-kB-NLRP3 axis.^[56]^ Overall, NF-kB is an important signaling pathway of inflammation and apoptosis in IVDD. It was previously found that after NPCs were stimulated by hypoxia and serum deprivation, ER regulates the homeostasis of the cell microenvironment through ER stress response, which is closely related to apoptosis, autophagy and senescence. It was shown that ERS activation during IVDD can increase the apoptosis of NPCs, promote the degradation of the extracellular matrix, and positively regulate autophagy in NPCs.^[57]^ Moreover, additional studies found that SIRT1 inhibited apoptosis by promoting the autophagic flux of human NPCs at the critical stage of degeneration.^[58]^

Although we have thoroughly demonstrated the mechanism of Duhuo in treating IVDD, this study still has several limitations. It relies heavily on computer simulation predictions (lacking laboratory validation) and depends on the integrity and quality of databases. Disease-gene annotation bias may exist, and some GEO datasets have small sample sizes (e.g., the comparison of 5 etc 5 samples in GSE56081). Furthermore, the active components of Duhuo and its regulatory mechanisms on key targets require further validation through in vitro and in vivo experiments.

In conclusion, the apoptosis of NPCs plays an important role in the process of IVDD, and is also closely related to autophagy and oxidative stress. We preliminarily found that the main components of Duhuo can regulate apoptosis and autophagy through IL-1β, TNF-a and BCL2, CASP3, CASP8, CASP9, and other targets in the NP. However, the specific mechanism needs to be confirmed by further experimental verification.

## 5. Conclusion

In summary, we established an IVDD disease prediction model based on apoptosis and autophagy hub genes such as CASP3, CASP8, and BCL2. We found that the pathogenesis of IVDD patients is mainly related to apoptosis, autophagy, and PI3K-Akt. Signaling pathway, FoxO signaling pathway, NF-κB signaling pathway and other signaling pathways are abnormally expressed. We finally confirmed that Duhuo treats IVDD by inhibiting the production of pro-inflammatory factors in the intervertebral disc, apoptosis and autophagy of NPCs.

## Acknowledgments

We acknowledge and GEO database for providing their platforms and contributors for uploading their meaningful datasets.

## Author contributions

**Conceptualization:** Qian Yan, Zhiwei Xu.

**Data curation:** Lei Yang, Zhifa Li.

**Supervision:** Xiaofei Wu, Chen Jiang.

**Writing – original draft:** Fei Liu, Chi Zhang.

**Writing – review & editing:** Zhifa Li, Feng Chen.
























